# Hybrid magnetic resonance and optoacoustic tomography (MROT) for preclinical neuroimaging

**DOI:** 10.1038/s41377-022-01026-w

**Published:** 2022-11-24

**Authors:** Zhenyue Chen, Irmak Gezginer, Mark-Aurel Augath, Wuwei Ren, Yu-Hang Liu, Ruiqing Ni, Xosé Luís Deán-Ben, Daniel Razansky

**Affiliations:** 1 Institute for Biomedical Engineering and Institute of Pharmacology and Toxicology, Faculty of Medicine, University of Zurich, Zurich, Switzerland; 2 Institute for Biomedical Engineering, Department of Information Technology and Electrical Engineering, ETH Zurich, Zurich, Switzerland; 3 Zurich Neuroscience Center (ZNZ), Zurich, Switzerland

**Keywords:** Imaging and sensing, Photonic devices

## Abstract

Multi-modal imaging is essential for advancing our understanding of brain function and unraveling pathophysiological processes underlying neurological and psychiatric disorders. Magnetic resonance (MR) and optoacoustic (OA) imaging have been shown to provide highly complementary contrasts and capabilities for preclinical neuroimaging. True integration between these modalities can thus offer unprecedented capabilities for studying the rodent brain in action. We report on a hybrid magnetic resonance and optoacoustic tomography (MROT) system for concurrent noninvasive structural and functional imaging of the mouse brain. Volumetric OA tomography was designed as an insert into a high-field MR scanner by integrating a customized MR-compatible spherical transducer array, an illumination module, and a dedicated radiofrequency coil. A tailored data processing pipeline has been developed to mitigate signal crosstalk and accurately register image volumes acquired with T1-weighted, angiography, and blood oxygenation level-dependent (BOLD) sequences onto the corresponding vascular and oxygenation data recorded with the OA modality. We demonstrate the concurrent acquisition of dual-mode anatomical and angiographic brain images with the scanner, as well as real-time functional readings of multiple hemodynamic parameters from animals subjected to oxygenation stress. Our approach combines the functional and molecular imaging advantages of OA with the superb soft-tissue contrast of MR, further providing an excellent platform for cross-validation of functional readings by the two modalities.

## Abstract (web summary)

We report on a fully hybridized magnetic resonance and optoacoustic tomography (MROT) system for concurrent noninvasive structural and functional imaging of the murine brain.

## Graphical abstract

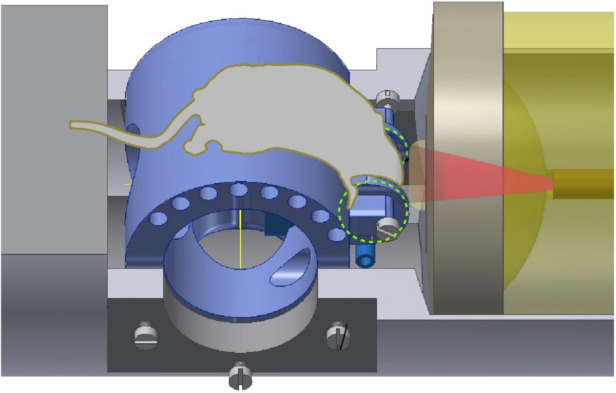

## Introduction

Small mammals, such as mice and rats, are the most prevalent animal models in neuroscience research owing to their good resemblance to many of the human brain functions^1^. Accessibility of genetic manipulations further allows for distinct labeling of neurons and other brain cells^2^, as well as developing models providing a better understanding of the molecular pathogenesis of Alzheimer’s, Parkinson’s, and other neurodegenerative diseases^3^. The excellent soft-tissue contrast provided by magnetic resonance imaging (MRI) has facilitated the widespread use of this method for mapping rodent brain anatomy^4^. Functional MRI (fMRI) has further become a powerful tool in neuroimaging because of its ability to detect hemodynamic changes associated with the resting-state and stimulus-evoked activity^5^. Common examples include functional connectivity studies of brain networks, optogenetic stimulation of specific cellular populations, chemogenetic stimulation of genetically-encoded receptors, or brain activity modulation with pharmacological agents^6^. However, specificity and sensitivity to hemodynamic changes achieved with blood oxygenation level-dependent (BOLD)-fMRI has remained a subject of substantial debate^7,8^ while researchers receding to alternative methods to characterize neural activity, e.g. via optical imaging techniques. The latter typically exhibit superior sensitivity to blood oxygenation yet suffer from low penetration and reduced spatial resolution in scattering tissues at depths beyond the ballistic regime of light propagation^9^.

In the last decade, optoacoustic tomography (OAT) has become a powerful neuroimaging tool bridging the gap between microscopic and macroscopic optical imaging modalities while benefiting from relatively high spatial resolution (<200 μm) in deep tissues and ultrafast volumetric imaging rates exceeding 1000 frames/sec^10^. OAT benefits from the unique advantages of optical contrast to visualize endogenous chromophores as well as exogenous absorbers, such as nanoparticles, organic dyes, or fluorescent proteins^11–13^. The method’s versatility was demonstrated in the label-free mapping of angiogenesis^14^ and blood oxygenation^15–18^, targeted molecular imaging studies^19–21^, or monitoring of therapies^22,23^, to name a few examples. Despite its important advantages, OAT often provides insufficient soft-tissue contrast, complicating anatomical interpretation of the data. Due to the inherent hybrid optical and ultrasound nature of OAT, multi-modality efforts have thus far been focused on hybridization with ultrasound^24–26^, fluorescence imaging^18,27–29^, or optical coherence tomography (OCT)^30–32^. Registration of OAT images with stand-alone computerized tomography (CT), single-photon emission computerized tomography (SPECT), and MRI has also been reported^4,33,34^.

**Fig. 1:**
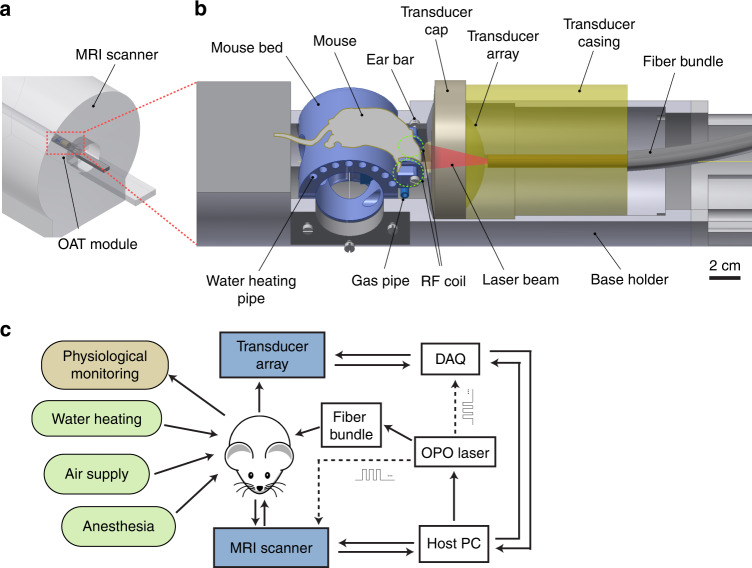
The hybrid magnetic resonance and optoacoustic tomography (MROT) scanner. **a** General system layout. **b** Detailed layout of the OAT module. **c** Block diagram of the different instrumentation used for in vivo measurements

Herein, we present a hybrid magnetic resonance and optoacoustic tomography (MROT) system for small animal studies. The OAT system was designed as a module to be inserted into a high-field MRI scanner (Fig. 1a). It integrates a customized MRI-compatible transducer array, an illumination module, and a dedicated radiofrequency (RF) coil. A tailored OAT data processing pipeline was also developed to reduce the interference from MRI and an image processing pipeline is proposed to facilitate data coregistration between the two modalities. We subsequently demonstrate concurrent acquisition of dual-mode anatomical and angiographic brain images with the scanner as well as real-time functional readings of multiple hemodynamic parameters from animals subjected to oxygenation stress.

## Results

### Hybrid MROT imaging

The MROT imaging system consists of a customized acoustically-coupled MRI-compatible transducer array, an illumination module, and animal fixation parts (see Methods for a detailed description). Due to the limited space inside the 9.4 T preclinical Bruker MRI scanner (Fig. 1a), all components in the OAT module are designed to fit within the 112 mm inner diameter bore while the animal is placed in a prone position on a cylindrically-shaped platform with the nose facing downwards (Fig. 1b). Two loops of saddle-shaped customized RF coils were integrated on either side of the mouse head holder to generate a magnetic field perpendicular to the main field, as required for MRI acquisitions (see Methods for details). Warm water was provided through polypropylene tubes inserted in the mouse bed to maintain constant animal body temperature (Fig. 1b). A polypropylene tube was also used to maintain anesthesia and subject the mouse to oxygen challenges (Fig. 1c, see Methods for details). The physiological status, including body temperature and breathing rate, was monitored in real-time with a small-animal physiological monitoring system (Fig. 1c, see Methods for a detailed description). The custom-made MR-compatible spherical matrix transducer array used for OAT image acquisitions consists of 384 trapezoidal elements with a 5 MHz central frequency. It achieves a nearly isotropic spatial resolution of 163.5 and 163.2 μm in the lateral and axial dimensions, respectively, as characterized by imaging a cluster of absorbing microspheres (Fig. S1, see Methods for a detailed description).

### Crosstalk analysis

**Fig. 2:**
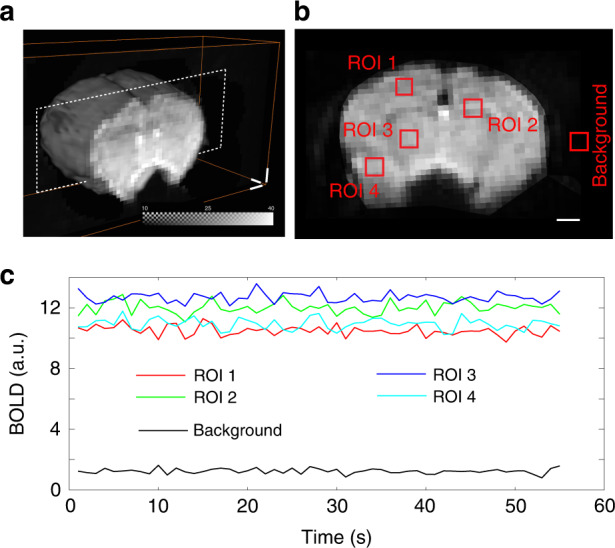
fMRI imaging in the presence of the OAT module. **a** Volumetric view of the mouse brain acquired with the gradient-echo echo-planar imaging (GE-EPI) sequence and simultaneous OAT. **b** Slice image of the mouse brain indicated by the dashed rectangle in (a). ROI size: 0.75 mm^3^ × 0.75 mm^3^ × 0.7 mm^3^. **c** Time course of the BOLD signal for the ROIs indicated in (**b**). All scale bars: 1 mm

Crosstalk between OAT and MRI is inevitable, as the strong electromagnetic interference caused by transient RF fields and magnetic gradients inside the 9.4 T scanner bore is picked up by the transducer cables^35^. On the other hand, the OAT scanning head, along with the coupling medium (water and ultrasound gel), may cause magnetic field distortions. These effects were minimized by employing a customized MR-compatible transducer, plastic casing for the illumination bundle, and heavy water (deuterium oxide) as the coupling medium (see Methods for details). In this manner, negligible image artefacts associated with magnetic field distortion were observed for different MRI sequences, including the EPI sequence known to be particularly susceptible to external interferences (Fig. 2). In vivo brain imaging using gradient-echo echo-planar-imaging (GE-EPI) sequence in the presence of the OAT module results in clearly resolvable brain surface mesh (Fig. 2a) and inner structures (Fig. 2b). The potential interference caused by OAT operation in a sequence of fMRI images was assessed by simultaneously recording blood oxygen level-dependent (BOLD) signals in vivo under resting-state conditions. As expected, the time course of the BOLD signal from different regions of interest (ROIs) indicated in Fig. 2b showed no significant spikes or other artefacts (Fig. 2c).

**Fig. 3:**
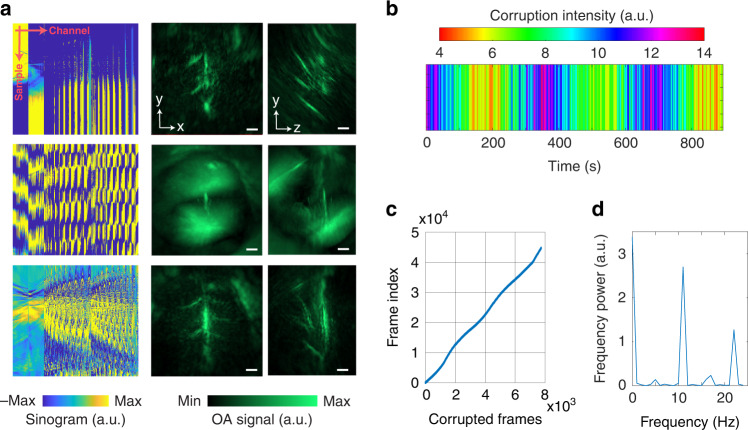
OAT signal corruption during concurrent fMRI scanning. **a** Sinograms (left) and corresponding OAT images of selected frames. Each sinogram is comprised of 384 transducer channels and 494 temporal samples. OAT images are shown as maximum intensity projection (MIP) in x-y (middle) and y-z (right) views. Scale bars: 1 mm. **b** Sinogram corruption intensity map during functional imaging. **c** Time series corrupted frame indices and **d** Corresponding corruption power spectrum by applying Fourier Transformation to (**c**). The peak frequency of 11 Hz corresponded to the slice number in one BOLD volume (i.e., RF pulse frequency). The laser pulse repetition frequency (PRF) was set to 50 Hz. Scale bars: 1 mm

In contrast, the OAT raw signals were strongly affected by the concurrent MRI scans mainly due to the RF pulses. Signal corruption was distributed across different time instances and transducer channels, as observed in the sinograms (Fig. 3a, left column). Thereby, direct image reconstruction with the raw signals results in corrupted OAT frames hindering subsequent data analysis (Fig. 3a, right column). To address this issue, we developed a sinogram corruption detection algorithm that calculates signal residuals after subtracting the nominal mean values of each channel (see methods for details, Fig. S2). If the L1 norm of the residuals from a sinogram exceeded a certain threshold, the sinogram was regarded as corrupted. Subsequently, a temporal corruption intensity map was calculated as the number of corrupted frames per second (Fig. 3b) based on the corrupted frame indices in the time-lapse image series (Fig. 3c). Furthermore, the data corruption power spectrum was obtained by performing Fourier analysis on the time course of corrupted frames (Fig. 3d). Note that the peak frame corruption frequency is corresponding to the slice number within a single BOLD volume. Adjusting the OAT data acquisition to occur precisely in the gap between the RF pulses is challenging since sweeping the laser with unequal time intervals requires perfect synchronization between the pump and laser emission output. Since individual RF pulses cannot be triggered for MRI data acquisition, a spontaneous temporal drift of those pulses inevitably introduces additional synchronization mismatches. In the second step, sinogram restoration was performed prior to image reconstruction. OAT sinograms corresponding to different laser excitation wavelengths were first separated. For each wavelength, the sinograms were downsampled and pixel-wise processing was performed to form new sinograms while rejecting outliers prior to frame averaging (see Methods for details).

### OAT image reconstruction

**Fig. 4:**
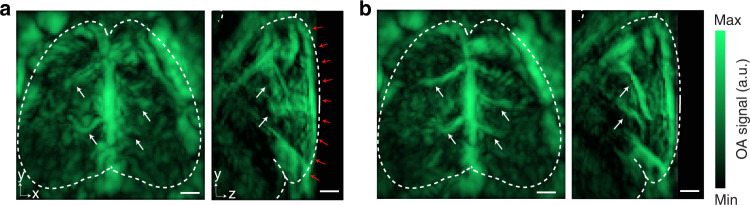
OAT image quality enhancement via reconstruction with the double speed of sound (SOS) approach accounting for the presence of acoustically mismatched heavy water. **a** Reconstructed OAT image at 800 nm considering a uniform SOS of 1400 m s^−1^. Images are shown as MIP in both x-y and y-z views. Red arrows in the y-z view depict the heavy water interface. **b** Corresponding reconstructed OAT image by considering SOS values of 1400 m s^−1^ for heavy water and of 1530 m s^−1^ for the mouse brain tissue. Structures that are better defined in (**b**) are indicated with white arrows. Scale bars: 1 mm

After sinogram restoration, OAT image reconstruction was first performed with a filtered back-projection algorithm^36^. The significant speed of sound (SOS) difference between heavy water and soft biological tissues has led to significant blurring and image distortion when assuming a uniform SOS for the reconstructions (Fig. 4a). In contrast, the iterative double SOS image reconstruction algorithm can achieve optimized performance to reveal sharper structures across the imaged volume (Fig. 4b, see Methods for details)^37^.

### OAT and MRI image coregistration

**Fig. 5:**
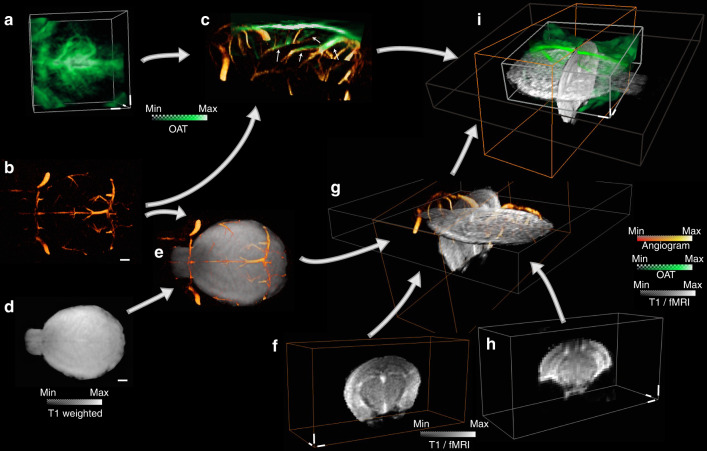
Data coregistration pipeline between OAT and fMRI images. **a** Reconstructed OAT image at 800 nm wavelength. **b** Magnetic resonance angiography (MRA) image. **c** Coregistration between OAT and MRA. **d** T1-weighted scan of the same mouse brain from the transverse view. **e** Coregistration between T1-weighted scan and MRA. **f** T1-weighted scan from the coronal view. **g** Coregistration between the two T1-weighted scans and MRA. **h** BOLD-fMRI image volume. **i** Coregistration between all the images acquired with the different OA and MRI sequences. Scale bars: 1 mm

Coregistration between OAT and MRI images is nontrivial due to the different contrast mechanisms provided by these modalities and the lack of common visible structures. In response, we developed a semi-automatic image registration protocol between OAT and MRI sequences using the SPM12 software^38^ (Fig. 5). OAT images mainly depict relatively large vessels, owing to the strong light absorption by whole blood (hemoglobin) relative to surrounding tissues (Fig. 5a). The vessels can therefore be exploited as landmarks (fiducial markers) for coregistration with magnetic resonance angiograms (MRA) (Fig. 5b) acquired with a fast low angle shot (FLASH) sequence. Given the 3D images simultaneously acquired by both modalities, the OAT images can be perfectly aligned to the MRA images using a simple rigid transformation without accounting for additional distortions (Fig. 5c). To facilitate coregistration between MRA and fMRI scans, two T1-weighted scans from the transverse and coronal views were performed (Fig. 5d, f). Since the MRA and the T1-weighted image from the transverse view (Fig. 5d) were acquired with the same modality, they can be readily registered (Fig. 5e). Subsequently, the T1-weighted scan from the coronal view (Fig. 5f) could be precisely aligned to the T1-weighted scan from the transverse view (Fig. 5d) with SPM12 based on the mutual information (Fig. 5g). Finally, fMRI data (Fig. 5h) was coregistered with OAT volumes to facilitate subsequent functional data analysis (Fig. 5i).

### Simultaneous dual-modality in vivo brain imaging under oxygen challenge

**Fig. 6:**
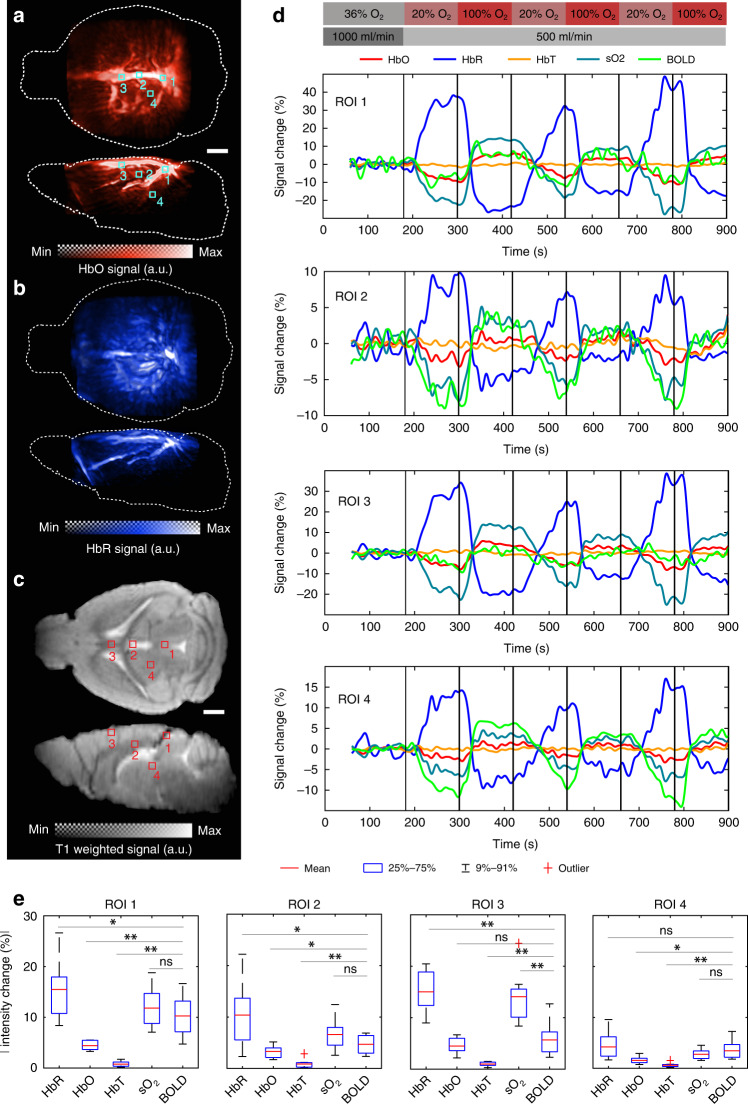
Simultaneous dual-modality in vivo brain imaging under oxygen challenge. **a**, **b** Oxygenated (HbO) and deoxygenated (HbR) hemoglobin components unmixed from the multi-wavelength OAT images. Maximum intensity projections (MIPs) from transverse and sagittal views are shown. **c** Simultaneously acquired T1-weighted MRI images. **d** Signal time courses from regions of interest in response to the breathing gas challenge paradigm. The ROIs (0.4 mm × 0.4 mm × 0.4 mm) are indicated in (**a**) and (**b**). **e** Statistics on absolute fractional signal changes in the different hemodynamic components across three mice, each receiving three stimulation cycles. The breathing gas stimulation paradigm is schematically depicted at the top of the panel (**d**) and elaborated in the methods section. *p < 0.05, **p < 0.01, ns not significant. Scale bars: 1 mm

The performance of the hybrid MROT system was further shown by noninvasive functional imaging of the mouse brain responses under a breathing gas challenge paradigm (Fig. 6). For this, the OAT data acquired at multiple wavelengths (see Methods) was unmixed into separate oxygenated (HbO) and deoxygenated (HbR) hemoglobin components^18^ (Fig. 6a, b) and coregistered to the MRI scans (Fig. 6c) following the protocol shown in Fig. 5. As expected, due to the strong light attenuation with depth, mainly superficial structures are emphasized in the OAT images whereas the MRI contrast is better balanced across the entire brain, further providing valuable anatomical reference information for OAT. On the other hand, OAT exhibited superior sensitivity to the hemodynamic changes generated by the oxygen challenge and was able to resolve multiple individual hemodynamic components undiscernible with BOLD-fMRI (Fig. 6d). Responses to the gas stimulation from the concurrent measurement were examined by selecting four distinctive ROIs in the major vessels and brain parenchyma. Interestingly, the signal profiles from all ROIs exhibited a decline in the HbO/sO_2_/BOLD levels and increased HbR during the transition from the baseline (36% O_2_) to normoxia (20% O_2_). A similar magnitude of responses was observed after a switch from normoxia to hyperoxia and vice versa, even though the amplitude of fractional changes varied from region to region. Statistics from the cohort study (*N* = 3, 9 stimulation cycles in total) revealed that the response amplitudes of HbR/HbO/sO_2_/BOLD were higher in major venous vessels (i.e., ROI 1 and 3, Fig. 6e) as compared to the brain tissue (i.e., ROI 2 and 4, Fig. 6e). These observations are generally consistent with previously reported observations under normobaric hyperoxic conditions, where an increase in venous oxygenation concentration over normoxic values of up to 10% was observed in BOLD-fMRI studies^39^. Although BOLD signals greatly correlate with HbR, their amplitudes are not linearly proportional to HbR changes across the ROIs, underscoring the complex nature of the BOLD signal ambiguously dependent on multiple hemodynamic components^8^. Moreover, depending on the ROI selected in the brain, each of the HbR/HbO/sO_2_ signals may have a different contribution to the overall BOLD signal (Fig. 6e). Interestingly, no significant change in HbT was observed during the entire oxygenation experiment across different ROIs.

## Discussion

The hybrid MROT system developed in this work enabled the first concurrent dual-modality observation of stimulus-evoked hemodynamic changes in the mouse brain. Hybridization of OAT and MRI is a particularly powerful multi-modal approach due to its important complementary advantages. First, it enables validation of dynamic brain responses recorded by OAT with the well-established fMRI modality, which further provides valuable anatomical landmarks due to its excellent soft-tissue contrast^40^. On the one hand, the relatively low temporal resolution of fMRI can be compensated with the very fast frame rates attainable with OAT^10,28^. Both modalities provide comparable spatial resolution and cover similar volumetric regions, further strengthening their complementary value. Finally, hemodynamic responses in the brain or other organs are dependent on multiple factors, such as blood flow, blood volume, the metabolic rate of oxygen consumption, and baseline physiological state^8^. OAT provides a unique capability to directly measure the bio-distributions of HbO and HbR and how these change in response to different types of stimuli^41^. The enhanced information enabled with simultaneous multi-contrast MROT acquisitions is then poised to shed light on the basic mechanisms underlying hemodynamic changes e.g., associated with neuronal activity.

Efficient hybridization between OAT and MRI for concurrent image acquisition has been a challenging technical task. One major issue is the limited space available in the typical preclinical MRI scanners due to the small bore diameter. The 9.4 T MRI scanner (BioSpec 94/20, Bruker BioSpin, Germany) used in this work has an inner bore diameter of 11.2 cm^42^, which is close to the 7–11 cm diameter of the matric array transducers commonly used for volumetric OAT imaging of the whole mouse brain^10^. This imposes strict limitations on the orientation of the transducer and the mouse head with respect to the RF coil used for the MRI data collection. The OAT module and mouse holder were specifically devised and 3D printed for imaging the mouse brain, thus a major redesign might be necessary for imaging other animals or other parts of the mouse body. The high static magnetic fields and strong RF gradients and pulses inside typical small animal MRI scanners represent another key limitation. On the one hand, all components of the OAT module must be customized based on non-magnetic materials. This is particularly relevant for the casing of the US array and the light guiding element. Furthermore, a large amount of water, which is placed close to the mouse head for acoustic coupling purposes, may have substantial negative effects on the loading of the close-by RF coil, shimming as well as wrap-around effects in the phase encoding direction. Hence, we opted for using heavy water (deuterium oxide, D_2_O) as an acoustic coupling medium to avoid local magnetic field distortions, which further necessitated a dedicated image reconstruction approach for OAT based on differential values of speed of sound in the medium. While no obvious distortion in the MRI images was observed due to the presence of the OAT module, the OAT images were strongly affected by electromagnetic interference from the RF gradients and pulses. This effect was reduced via synchronization between OAT and MRI trigger signals and rejection of affected frames in post-processing. To facilitate accurate coregistration between the images acquired by the two modalities, a 3D-printed holder was designed to stereotactically fix the mouse head with two customized RF coils integrated into the holder so that the same volume was effectively covered with both OAT and MRI.

In the current system implementation, a comparable spatial resolution in the 200μm range was achieved with both modalities, while the volumetric imaging rate was significantly higher for OAT (up to 100 Hz, limited by the PRF of the OPO laser) than for MRI (up to ~2 Hz for an adequate SNR). The spatio-temporal resolution performance of MROT can potentially be enhanced. For example, state-of-the-art MRI equipment has enabled reaching a temporal resolution of less than 20 ms with high-speed techniques such as FLASH with gradient echo^43,44^. Other MRI methods have also achieved real-time imaging performance to the detriment of spatial resolution or by opting for 2D slice imaging instead of volumetric acquisitions^45^. Also, spatial resolution down to 30 μm has also been achieved with ultrahigh magnetic fields^42^. The MRA contrast and resolution can be enhanced via injection of a blood pool and long-circulating contrast agents providing increased contrast from small vessels^46^. New methods have also been reported for enhancing the spatio-temporal resolution of OAT e.g. based on sparse acquisitions^47^, regularized iterative inversion of a broadband US model^48^, or localization and tracking of particles^49^. Also important is the fact that MROT can further be hybridized with other imaging modalities. For example, the integration of a fluorescence macroscopic imaging system can open opportunities for exploiting the contrast provided by a wide range of fluorescent sensors, such as genetically-encoded calcium indicators^28^ that can be used to visualize neural activity directly. OAT has also been combined with functional US^50^, the latter additionally providing blood flow information^51^.

In conclusion, the proposed integrative MROT imaging approach can be used to expand our understanding of spontaneous and stimulus-evoked brain activity, neurovascular coupling mechanisms, and brain metabolism. Both OAT and MRI can capture the induced hemodynamics responses in the entire mouse brain, providing highly complementary concurrent readings that can potentially be used to generate fundamentally new insights into brain activity and neurological and neurodegenerative disorders.

## Materials and methods

### MROT imaging system

The hybrid MROT system is based on a high-field MRI scanner (BioSpec 94/20, Bruker BioSpin, Germany, Fig. 1a). The OAT module integrates a customized MRI-compatible transducer array (Imasonic SAS, France), an MR-compatible fiber bundle (CeramOptec GmbH, Germany) and a customized RF coil. The transducer array consists of 384 trapezoidal elements with 5 MHz central frequency and >80% detection bandwidth distributed on a hemisphere with a radius of 40 mm and angular aperture of 130° (1.48π solid angle). It was confined within a cylindrical volume with a diameter of 90 mm and length of 110 mm to fit within the limited space inside the 112 mm diameter MRI scanner bore. For OAT brain imaging, the animal was placed in its prone position with the nose facing down so that the brain lay within the FOV covered by the OAT probe. Acoustic coupling was ensured with deuterium oxide (heavy water) sealed within a customized non-magnetic polyetheretherketone (PEEK) cap attached to the transducer array (Fig. 1b). A 36 mm central opening in the cap was pre-sealed with a thin polyethylene layer transparent for both light and ultrasound waves within the detection bandwidth of the array. OA signal excitation was performed with a short-pulsed (<10 ns) tunable optical parametric oscillator (OPO) laser (Spit-Light, Innolas Laser GmbH, Germany) guided through a central 8 mm aperture in the transducer array by means of the custom-made fiber bundle (Fig. 1b). The measured pulse energy at the output of the illumination fiber bundle was ~8 mJ. The OA signals collected by the array elements were digitized with a custom-made data acquisition system (DAQ, Falkenstein Mikrosysteme GmbH, Germany) at 40 mega samples per second (Msps). The recorded raw data were eventually transmitted to a PC via Ethernet. MRI was performed with a pair of customized RF coils integrated on both sides of the mouse head holder to generate a magnetic field perpendicular to the main magnetic field of the scanner (Fig. 1b). MROT data acquisition was triggered with the Q-switch output of the laser and synchronized with an external trigger device (Pulse Pal V2, Sanworks, USA).

### Spatial resolution characterization

The spatial resolution of the OAT system was characterized by imaging a cluster of microspheres (Cospheric, USA) with diameters ranging between 38 to 42 μm embedded in an agar phantom. For this, the laser wavelength was tuned to 800 nm. Two hundred frames were acquired and averaged for OAT image reconstruction (Fig. S1a). A microsphere close to the center of the FOV was selected to extract lateral (Fig. S1b, d) and axial (Fig. S1c, e) image profiles. The width of these profiles was quantified as the full width at half maxima (FWHM) of the fitted Gaussian curves. Considering the small diameter of the sphere, these width values were considered to represent a good estimate of the spatial resolution. In MRI, the spatial resolution is principally determined by the voxel size of the volumetric image, which greatly varies for the different sequences depending on the selected matrix size, FOV, and slice thickness, as described in the MRI data acquisition section.

### Animal models

Athymic female nude mice (Foxn1^nu^, Charles River Laboratories, USA, 6-week-old, *N* = 3) were imaged in this study. The animals were housed in individually ventilated, temperature-controlled cages under a 12-h reversed dark/light cycle. Pelleted food (3437PXL15, CARGILL) and water were provided ad libitum. Mouse housing, handling, and experimentation were performed in accordance with the Swiss Federal Act on Animal Protection and were approved by the Cantonal Veterinary Office Zurich (license #ZH161/18).

### In vivo animal experiments

Mice were anesthetized with an oxygen/air mixture (200:800 mL/min) in an induction box (4% isoflurane), and anesthesia was subsequently supplied through a breathing mask (1.5% isoflurane). In vivo imaging was performed by placing each mouse onto the 3D-printed mouse bed in a prone position with the nose pointing downwards. Ultrasound gel mixed with heavy water was applied on the mouse head to ensure optimal acoustic coupling through the transparent polyethylene membrane while minimizing the signal distortion in MRI data acquired with different sequences. The mouse head was immobilized using a custom 3D-printed stereotactic frame. After positioning the mouse, the platform containing both the ultrasound array and the RF coil was inserted into the bore of the MRI scanner. The acquisition was initiated after the physiological status of the mouse remained stable for at least 10 min. A 15 min duration breathing gas challenge paradigm was adopted consisting of the following steps. For the first 3 min, mixed air (oxygen/air 200:800 mL/min, FiO_2_ = 36%) was supplied as a baseline. The subsequent hyperoxia challenge consisted of three cycles. In each cycle, normoxia (oxygen/air 0:500 mL/min, FiO_2_ = 20%, 2 min) and hyperoxia (oxygen/air 500:0 mL/min, FiO_2_ = 100%, 2 min) was induced. For OAT data acquisition, the laser wavelength was rapidly swept between five wavelengths (700, 730, 755, 800, and 850 nm) on a per-pulse basis at 50 Hz pulse repetition frequency (PRF). The measured pulse energy at the output end of the fiber bundle was ~11 mJ. fMRI data were simultaneously acquired using a GE-EPI sequence^52^. Other sequences, such as T1-weighted and 2D FLASH angiography, were also performed to facilitate data coregistration. Detailed parameters are provided in the MRI data acquisition section.

During the experiment, the physiological status of the animal was monitored in real-time. Specifically, the body temperature was measured with a rectal thermometer, while the respiration was measured with a pneumatic pillow (SA Instruments, USA). The heart rate and peripheral oxygen saturation (SpO_2_) were also monitored in real-time with an MR-compatible mouse paw pulse oximeter (PhysioSuite, Kent Scientific Corporation, USA). The animal was kept warm with a tunable water heating unit and the body temperature was maintained at ~37 °C.

### MRI data acquisition

MRI data acquisition was conducted on a 9.4 T Bruker Biospec 94/20 small animal MRI system (Bruker BioSpin MRI, Ettlingen, Germany) using custom-made RF coils. ParaVision 6.0.1 was used as the user interface. MRA images were acquired to facilitate image coregistration with OAT using a FLASH sequence: FOV = 20 mm^2^ × 20 mm^2^, matrix dimension (MD) = 256 × 256, 20 slices from the brain surface to deeper regions, slice thickness = 0.3 mm, repetition time (TR) = 13 ms, echo time (TE) = 1.8904 ms, number of averages (NA) = 4. A corresponding T1-weighted scan in the transverse plane serving as the anatomical reference was acquired using a FLASH sequence: FOV = 20 mm^2^ × 20 mm^2^, MD = 128 × 128 × 20, 20 slices from the brain surface to deeper regions, slice thickness = 0.3 mm, TR = 500 ms, TE = 2.6203 ms, NA = 6. Subsequently, another T1-weighted scan in the coronal plane serving as the anatomical reference to the fMRI data was acquired using the FLASH sequence: FOV = 20 mm^2^ × 10 mm^2^, MD = 160 × 80, 11 slices, slice thickness = 0.7 mm, TR = 500 ms, TE = 2.1366 ms, NA = 8. Note that prior to fMRI data acquisition, the local field homogeneity was optimized using acquired field maps. BOLD-fMRI images were acquired using a GE-EPI sequence: FOV = 20 mm^2^ × 10 mm^2^, MD = 80 × 40, yielding an in-plane voxel dimension of 250 × 250 µm^2^, 11 slices, slice thickness = 0.7 mm, flip angle (FA) = 60 °, TR = 995 ms, TE = 12 ms, NA = 1, and a temporal resolution of 1 s per image data set. For the oxygen challenge paradigm, the scan length was set to 900 repetitions.

### OAT image reconstruction

For OAT image reconstruction, the raw signal matrices were first separated by wavelength. For each wavelength, signal corruption analysis was performed with a sinogram corruption detection algorithm by calculating the residuals after subtracting the nominal mean values of each channel. If the L1 norm of the residuals exceeded a certain threshold, the sinogram was regarded as corrupted. A sinogram restoration was subsequently performed (Fig. 3). The sinograms were downsampled to 1 Hz to match the fMRI volume rate. In this step, pixel-wise processing was performed to form the new sinogram, and outliers from ten adjacent measurements were rejected before averaging. After sinogram restoration, image reconstruction was implemented with a single SOS at 1400 m s^−1^ such that superficial vessels are sharply defined. This facilitates segmenting the interface between heavy water medium and the skin surface^36^. Specifically, 3D segmentation was performed by considering a simple geometry. Finally, a filtered back-projection algorithm^37^ (bandpass filter 0.1–8 MHz) was employed for OAT image reconstruction considering two SOS values, i.e., 1400 m s^−1^ for heavy water medium and 1530 m s^−1^ for the brain tissue. To reduce the computational burden, a voxel size of 100 μm^3^ × 100 μm^3^ × 100 μm^3^ and FOV of 8 mm^3^ ×8 mm^3^ × 4 mm^3^ were chosen. During reconstruction, OAT signals were normalized to the laser pulse energy at each wavelength. To reduce the spectral coloring effects, i.e., the spatially and wavelength-dependent light fluence distribution within the tissue, an exponential light attenuation model was applied to compensate for the depth-dependent signal decay^18^. Finally, linear spectral unmixing was performed to directly obtain the individual HbO and HbR components without additional processing^18,53^. The data processing, including OAT image reconstruction and spectral unmixing, was automated in a customized script written in Matlab (version 2021a, Mathworks Inc., Natick, MA, USA). The processing time is roughly 30 min for a dataset comprising of 45,000 volumetric image frames with a PC equipped with a 6-core AMD Ryzen 5 2600X processor and NVIDIA GeForce GTX1060 graphics card.

### Functional data analysis

Functional data analysis was performed using Matlab. Before data analysis, other hemodynamic (derivative) components, such as HbT and sO_2_, were first calculated voxel-wise via HbT = HbO + HbR and sO_2_ = HbO/HbT. Then, OAT and fMRI data were realigned, coregistered and resliced with the SPM12 software^38^. To show the responses to the breathing gas stimulation paradigm, different ROIs with a volume size of 0.4 mm × 0.4 mm × 0.4 mm were selected across the mouse brain from the multiparametric data acquired by OAT and fMRI. Fractional signal changes in each hemodynamic component were calculated by ΔS/S = (S(t) – BL)/BL where BL was calculated as the mean value during the first 3 min baseline recording. A Butterworth lowpass filter with a normalized half-power frequency of 0.15 was applied to each signal profile to display a smooth response curve. For statistics, the response amplitude of each hemodynamic component was calculated as the maximum absolute fractional signal change in each stimulation cycle for all three mice, each receiving three oxygen stimulation cycles. Mean values and their corresponding standard deviations were thus calculated from a group of nine data points for each component in each ROI. Data were shown with the boxplot function in Matlab.

### Statistical analysis

Statistical analysis was performed with Matlab. The significance of signal changes between HbR/HbO/HbT/sO_2_ and BOLD was tested with a two-sample two-tailed *t*-test across nine stimulation cycles from three mice. Statistical significance was set at *p* < 0.05.

## Supplementary information

Supplementary Information
