# Supplementary material for: Hybrid magnetic resonance and optoacoustic tomography (MROT) for preclinical neuroimaging
Source: Light Sci Appl. 2022 Nov 24;11:332. doi: 10.1038/s41377-022-01026-w (PMC9684112; doi:10.1038/s41377-022-01026-w)
Supplement: Supplementary file 1 — Supplementary Information [file 41377_2022_1026_MOESM1_ESM.docx]

**Supplementary Information for**

**Hybrid magnetic resonance and optoacoustic tomography (MROT) for preclinical neuroimaging**

Zhenyue Chen,^1,2^ Irmak Gezginer,^1,2^ Mark-Aurel Augath,^1,2^ Wuwei Ren,^1,2^ Yu-Hang Liu,^1,2^ Ruiqing Ni, ^1,2,3^ Xosé Luís Deán-Ben^1,2^, and Daniel Razansky^1,2,3,*^

^1^Institute for Biomedical Engineering and Institute of Pharmacology and Toxicology, Faculty of Medicine, University of Zurich, Switzerland

^2^Institute for Biomedical Engineering, Department of Information Technology and Electrical Engineering, ETH Zurich, Switzerland

^3^Zurich Neuroscience Center (ZNZ), Switzerland

*Correspondence

Daniel Razansky, Institute for Biomedical Engineering, Wolfgang-Pauli-Str. 27, 8093 Zurich, Switzerland

Email: [daniel.razansky@uzh.ch](mailto:daniel.razansky@uzh.ch)


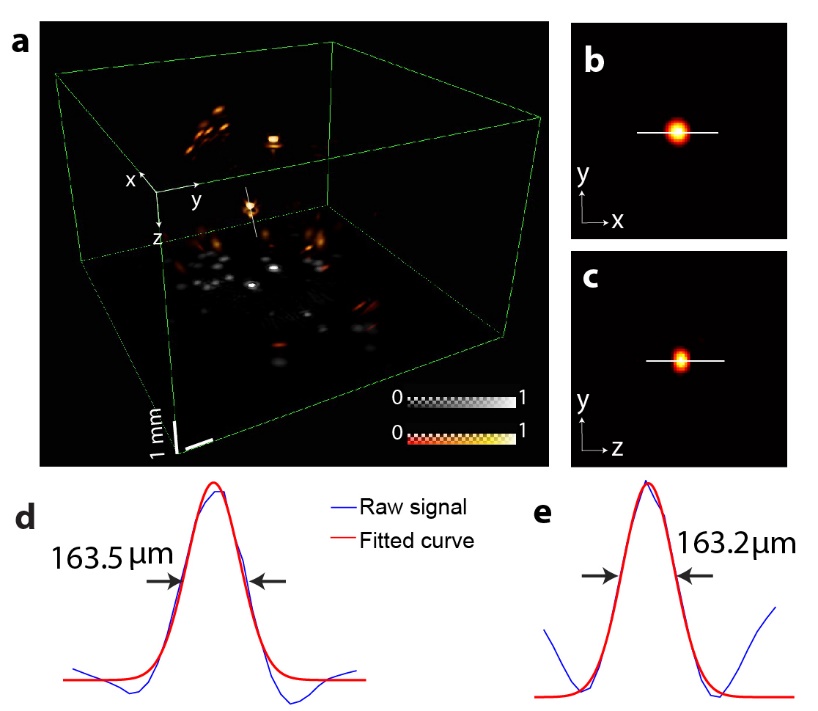


**Figure S1 |** Spatial resolution characterization of OAT by imaging a cluster of microspheres with diameters ranging between 38-42 μm. **a** Volumetric OAT image reconstructed from the microsphere phantom. **b**,**c** Slice image of the selected microsphere in x-y and y-z views, respectively. **d**,**e** One-dimensional signal profiles along the white lines indicated in **b** and **c**, depicting the lateral and axial resolutions of ~163.5 μm and ~163.2 μm, respectively.


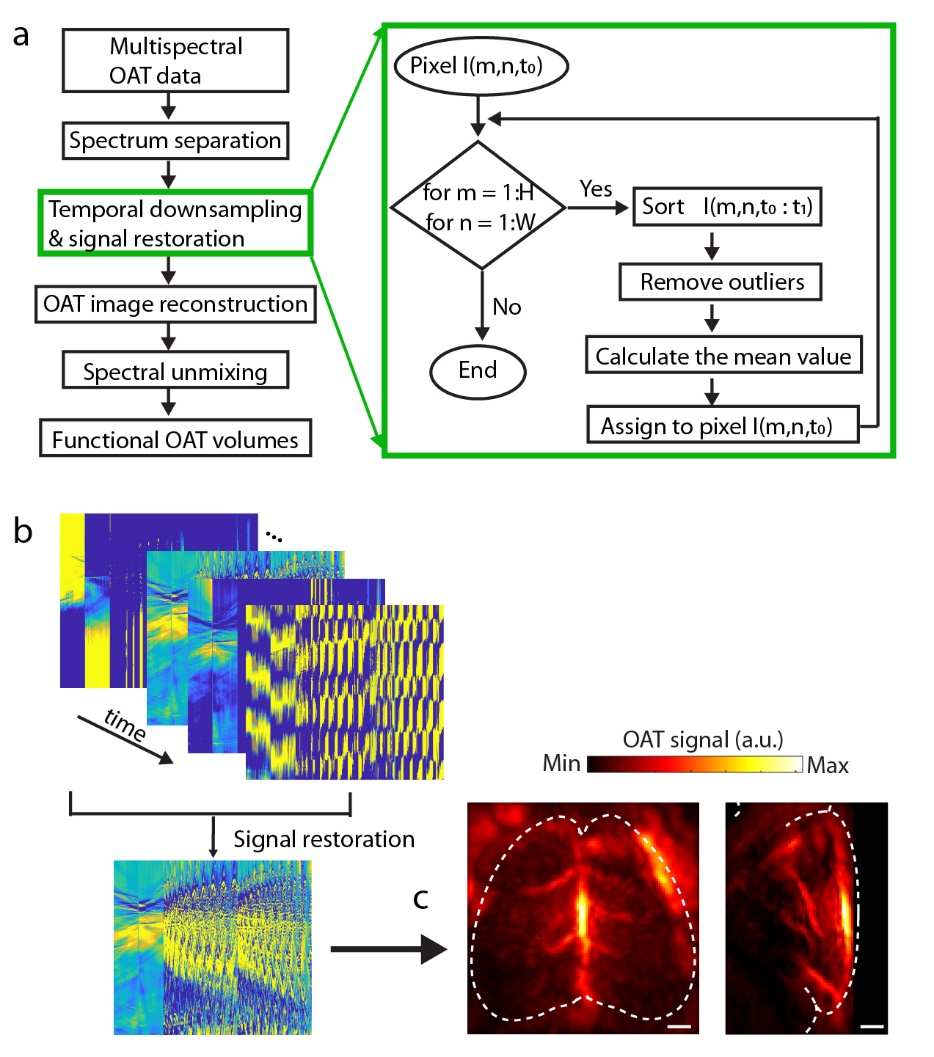


**Figure S2 |** OAT sinogram restoration and image reconstruction. **a** OAT image reconstruction pipeline. The sinogram restoration proceedure is shown in the green box. **b** Illustration of sinogram restoration and **c** corresponding OAT reconstruction shown in x-y and y-z views. Scale bar: 1 mm.
